# ‘Everyone has heard of it, but no one knows what it is’: a qualitative study of patient understandings and experiences of herpes zoster

**DOI:** 10.3399/BJGP.2024.0025

**Published:** 2025-01-14

**Authors:** Sophie Rees, Matthew Ridd, Lorelei Hunt, Hazel Everitt, Anna Gilbertson, Robert Johnson, Anthony E Pickering, Oliver van Hecke, Vikki Wylde, Sian Wells, Jonathan P Banks

**Affiliations:** Bristol Trials Centre, Population Health Sciences, University of Bristol, Bristol, UK.; Centre for Academic Primary Care, Population Health Sciences, University of Bristol, Bristol, UK.; Bath, UK.; School of Primary Care, Population Sciences and Medical Education, University of Southampton, Southampton, UK.; Centre for Academic Primary Care, Population Health Sciences, University of Bristol, Bristol, UK.; Translational Medicine, Faculty of Health Sciences, University of Bristol, Bristol, UK.; Anaesthesia, Pain and Critical Care Research, School of Physiology, Pharmacology and Neuroscience, Faculty of Life Sciences, University of Bristol, Bristol, UK.; Centre for General Practice, Department of Public Health and Primary Care, Ghent University, Ghent, Belgium.; Department of Public Health and Primary Care, Ghent University, Ghent, Belgium.; Bristol Trials Centre, Population Health Sciences, University of Bristol, Bristol, UK.; National Institute for Health and Care Research Applied Research Collaboration West, University Hospitals Bristol & Weston NHS Foundation Trust, Bristol, UK.

**Keywords:** general practice, pain, patient experience, qualitative research, shingles

## Abstract

**Background:**

Shingles (herpes zoster), caused by reactivation of the varicella-zoster virus, is usually diagnosed and managed in primary care. The lifetime risk of shingles in the general population is approximately 30%, and it can have a detrimental effect on quality of life. There has been little qualitative research about patient experience and understanding of shingles.

**Aim:**

To explore patient experiences and understanding of shingles.

**Design and setting:**

Qualitative interviews with people with shingles recruited from primary care in England.

**Method:**

Qualitative semi-structured remote interviews were undertaken with 29 participants in a randomised controlled trial in primary care in England (ATHENA, ISRCTN14490832). Participants were aged >49 years and were diagnosed within 6 days of shingles rash onset. Interviewees were sampled for diversity in terms of pain, intervention adherence, age, gender, and ethnicity. Data were analysed using reflexive thematic analysis.

**Results:**

Interviews took place in November 2022 to April 2023. Participants’ understanding of shingles was limited, particularly pre-diagnosis. A common theme was that ‘everyone has heard of it, but no one knows what it is’. Television campaigns about the shingles vaccination programme helped some to recognise the rash. Shingles was understood as a disease with a variable prognosis, resulting in a sense of uncertainty about the significance when diagnosed. Participants reported a range of symptoms, which impacted on everyday life. Some people thought their diagnosis was caused by poor mental health or challenging life circumstances, a perception sometimes reinforced by healthcare professionals. Many participants sought meaning in their diagnosis, reflecting on, and sometimes changing, their life and circumstances.

**Conclusion:**

Primary care practitioners should be aware of the broad spectrum of patient knowledge, and the potential for better understanding to promote early attendance and treatment to reduce the impact of shingles.

## Introduction

Herpes zoster, commonly known as shingles (and hereafter referred to as ‘shingles’), is caused by the reactivation of varicella zoster virus (chickenpox virus) in adults. The lifetime risk of shingles in the general population is approximately 30%, and this increases with age.^1^ The onset and subsequent symptoms of the condition are quite distinct. It is characterised initially by prodromal symptoms such as pain, tingling, headache, or malaise, usually followed by the onset of a painful rash, which can last for several weeks.^2^ Shingles can detrimentally affect health-related quality of life and functional status, impacting on sleep, enjoyment of life, and activities of daily living.^3^^–^5

However, there is limited evidence about how people understand and make sense of shingles. Analysis of patients’ perceptions could help address any misconceptions and facilitate better communication between patients and healthcare professionals. A Danish focus group study found that awareness of shingles was low, leading to delayed help seeking.^6^ Participants also felt that shingles was trivialised by others who had a poor understanding of the reality of the experience.^6^ A study in Germany,^7^ which used interviews and questionnaires, reported that attitudes towards shingles changed after diagnosis, from an idea of it as ‘harmless’ to ‘severe or very severe’. An interview study of patients in Canada found that shingles negatively impacted on many dimensions of health-related quality of life.^8^

Building on this limited evidence base, the aim of this study was to explore people’s lived experiences of shingles, and how they make sense of it.

**Table:** How this fits in

Limited literature about the experience and understanding of shingles suggests that people tend to think of it as minor until they experience it themselves. We found that shingles was not experienced as a minor illness, with the condition raising questions for participants about their age, lifestyle, and mental wellbeing. In interactions with patients, it is important that GPs and healthcare professionals recognise these potential sensitivities. Healthcare professionals and public health campaigns should ensure that people understand that they should not blame themselves for their illness or feel ashamed.

## Method

### Study design

This was a qualitative study embedded within the AmiTriptyline for the prevention of post-HErpetic NeuralgiA (ATHENA) trial (ISRCTN14490832), which is testing whether low-dose amitriptyline can prevent post-herpetic neuralgia in patients with shingles.^9^

The aims of the study were to assess intervention acceptability and perceived effectiveness, and to explore participant experiences of shingles. This article reports on the latter aim, with views on the intervention to be reported separately. Data were collected via semi-structured interviews and analysed using reflexive thematic analysis.^10^

### Recruitment and sampling

ATHENA trial participants were referred by a trained primary healthcare professional, after presenting to a participating GP surgery with shingles and confirmed as eligible for the trial. Consent was received and randomisation undertaken by a member of the research team, who also asked for permission to approach participants for an interview. Interviewees were ATHENA trial participants who were recruited 10– 12 weeks post-randomisation and after the primary outcome (prevalence of post-herpetic neuralgia) had been recorded. Participants were contacted by a member of the study team via email or telephone, provided with information, and given an opportunity to ask questions. Verbal audio-recorded informed consent was obtained at the start of each interview, and interviewees were offered a £10 high street shopping voucher.

We sampled for maximum variation by: age, gender, and ethnicity, and diversity of pain (measured by the Zoster Brief Pain Inventory^11^ at 30 days post-randomisation) and treatment adherence.

We aimed for 30 participants, evenly split between the two trial arms (amitriptyline and placebo), supported by an unmasked study statistician. This sample size was chosen to enable us to collect a manageable amount of rich data from the two trial arms. Based on previous experience, we estimated this sample size would be sufficient to answer our research questions, which also included acceptability and perceived effectiveness of the intervention (to be reported elsewhere). The qualitative team remained masked during data collection and analysis to avoid influencing participants or data interpretation. Data were reviewed regularly by the qualitative team to assess whether the data had sufficient information power^12^ to address the research questions.

### Data collection

We developed a topic guide as a team, refining and improving it in collaboration with the ATHENA patient and public involvement group. For example, they encouraged us to ask about ongoing symptoms other than pain, and helped to word a question about fears about long-term pain. Interviews were by remote audio-recorded telephone or video call. All interviews were conducted by one researcher.

### Analysis

Audio-recordings were transcribed verbatim, pseudonymised, and imported into NVivo (version 12) for analysis. Two authors each coded five transcripts and met to discuss and develop a flexible coding frame. Four clinical members of the trial management group also read one to three transcripts each and provided written comments on data to inform the coding frame. One researcher coded the remaining dataset, meeting regularly with another researcher to discuss ongoing analysis. The analysis evolved from the initial coding frame to the final themes, for example, ‘talking to others about shingles’ was part of the topic guide and initial coding frame, and through analysis this became ‘everyone has heard of it, but no one knows what it is’.

## Results

### Participants

Interviews took place from November 2022 to April 2023. We approached 47 trial participants, and 29 agreed to be interviewed. We reviewed the data regularly and judged we had sufficient information power at 29.^12^ Interviews lasted 30 minutes on average (range 15– 44 minutes). The characteristics of the sample are shown in Table 1.

**Table 1.** Participant characteristics

**Characteristic**	***n* (%)**
**Age, years**	
50–59	10 (34)
60–69	13 (45)
70–79	5 (17)
80–89	1 (3)

**Gender**	
Female	14 (48)
Male	15 (52)

**Ethnicity**	
White	26 (90)
Black/African/Caribbean/Black	1 (3)
British/Black other	2 (7)
Mixed/multiple ethnic groups	

**Trial arm**	
Amitriptyline	15 (52)
Placebo	14 (48)

**Pain at 30 days**	
None/mild (0–2 on the ZBPI)	22 (76)
Severe (3+ on the ZBPI)	6 (21)
No data	1 (3)

**Shingles vaccination status**	
Unvaccinated	26 (90)
Vaccinated	3 (10)

*ZBPI = Zoster Brief Pain Inventory. Response in the 90-day questionnaire to the question ‘Please rate your pain by choosing the one number that best describes your pain at its worst in the past 24 hours’ (0–10). A score of ≥3 on the ZBPI after 3 months is considered indicative of post-herpetic neuralgia.^11^*

**Figure 1.**
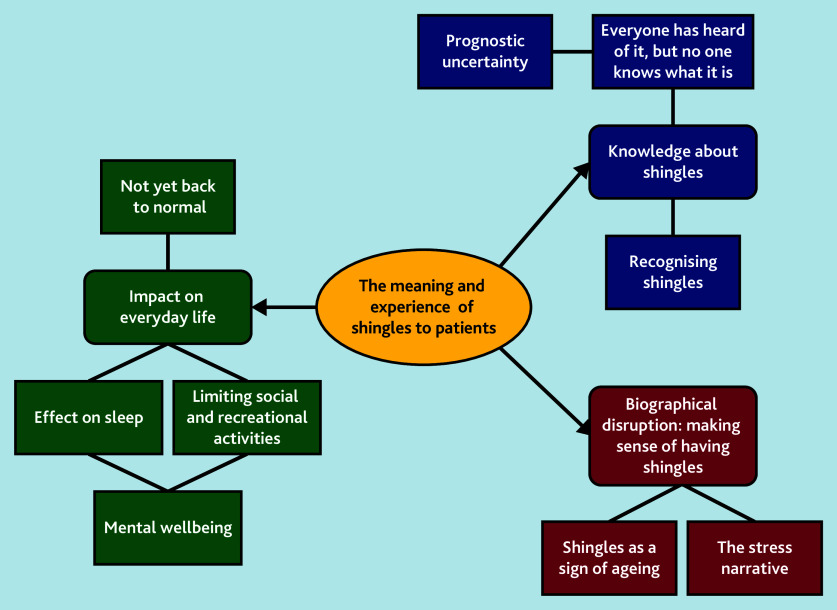
Summary of themes and subthemes.

Results are organised below under three broad themes: ‘knowledge about shingles’ looks at patient understanding of the condition from symptom onset to diagnosis; ‘biographical disruption’ looks at how patients make sense of their illness in the context of their lives; and ‘impact on everyday life’ looks at how symptoms limit patients’ ability to engage with work and social activities. Figure 1 summarises the themes and subthemes.

### Knowledge about shingles

#### Everyone has heard of it, but no one knows what it is

Despite being aware of the disease, most participants felt they knew very little about shingles before their diagnosis. Participants’ understanding of shingles was often vague and limited, and this was reflected through conversations in their wider social network:

*‘Everyone seems to have heard of shingles, but doesn’t* [*sic*] *know really what it is…’*

(ATHENA Qualitative ID (AQ)042, Male, 61 years old)

*‘I mentioned it to a few people and they all contradicted me. But I didn’t know that it was related to herpes, although I’ve since learned that it’s chickenpox.’*

(AQ058, Male, 59 years old)

Patient confusion about shingles was present in their understanding about the reoccurrence and the effectiveness of vaccines:

*‘I’ve had it already, I didn’t expect it could come back again.’*

(AQ043, Female, 63 years old)

*‘I was a bit disappointed because I had a shingles vaccination, so I didn't expect to get it … And it really infuriates me that* [pharmaceutical companies] *are advertising injections … because they don't always work.’*

(AQ047, Female, 72 years old)

As a result of lack of knowledge about shingles pre-diagnosis, participants felt unprepared for the pain and symptoms they experienced:

*‘It was incredibly painful. I couldn't rest, I couldn't sleep at night with it so I found it really, really distressing in a way that I hadn't expected it to be really. So, that was a bit of an eye opener and I suddenly thought, instead of thinking of shingles as this thing that old people get, I thought actually, this is really quite unpleasant.’*

(AQ061, Female, 57 years old)

#### Recognising shingles.

Typically, participants misattributed their prodromal (pre-rash) symptoms to another injury or illness:

*‘I just thought I had injured my shoulder because the pain was quite intense.’*

(AQ068, Male, 81 years old)

Previous experience of shingles, either personally or through others around them, helped participants to recognise it:

*‘I thought that I had some insect bites … on the front of my bust but after a day or two, I suddenly realised that they* [were] *beginning the characteristic blisters of shingles and I knew that because I had shingles probably about thirty years ago, so I knew what it looked like. I also realised there was a bit on my back as well and that it was half my body. And at that stage I phoned my GP that morning.’*

(AQ047, Female, 72 years old)

*‘Few red lumps come and that, particularly one right between the eyes, above the nose. Had to do a board meeting with it … somebody identified that they thought it was shingles. It was one of my staff. ’Cause we use Microsoft Teams, I’m chatting to him and we have the camera on. They said “Oh blimey you know,* [name]*, that’s shingles. I’ve had it before. Get up the doctor quick.”’*

(AQ045, Male, 63 years old)

Television campaigns about the shingles vaccination programme also helped participants recognise it:

*‘There’s just been adverts about it on the TV so and then when I had the pains and the blotches turned up, I'm thinking “oh, hang on”.’*

(AQ044, Female, 60 years old)

Primary care practitioners were reported to be quick at responding to a possible shingles diagnosis:

*‘My daughter sent a photo to the clinic and the doctors phoned me back practically straight away.’*

(AQ063, Female, 74 years old)

While all of the participants were diagnosed within 6 days of rash onset (to be eligible for the ATHENA trial), some participants reported prodromal symptoms, which were investigated pre-rash onset. For these participants, diagnosis felt delayed and drawn out:

*‘I had a pain around the right-hand side of my stomach … After a couple of days my family advised me to contact my surgery … They referred me to a physiotherapist … he treated me, manipulated me … I thought, “oh, it’s a little bit better, but there’s still a pain” … “I wonder if there might be something wrong with my gallbladder” … So, I had to contact my GP again. They made an appointment for me a few days after … On the day on my back there was some sort of itchiness. It wasn’t even itchy. Some sort of a spot … When I went to the doctor, the doctor asked me to show my back. And she knew straight away it was shingles.’*

(AQ043, Female, 63 years old)

The lack of knowledge about shingles among some participants led to delayed presentation. Where a participant was aware of shingles and mentioned this to their primary care provider, diagnosis was often much faster, highlighting the importance of awareness.

#### Prognostic uncertainty.

Participants experienced uncertainty following diagnosis because shingles is characterised by prognostic uncertainty:

*‘Shingles is not a particularly nice thing as you get older … It can affect you really badly. It’s a bit like COVID. You roll the dice when you get it, and wonder how it’s going to affect you. You don’t really know how it’s going to affect you until you have it.’*

(AQ049, Female, 64 years old)

This prognostic uncertainty was often evident when interviewees looked to the experiences of others in their social network, where the range of experiences was highly varied:

*‘People were saying, “Oh, it was awful” and some saying, “Well, it was like measles and nothing much.”’*

(AQ065, Male, 65 years old)

### Biographical disruption: making sense of having shingles

#### Shingles as a sign of ageing.

For many participants, shingles was viewed as a disease of the older person, and a reminder that they themselves are ageing, which had a direct impact on their sense of identity:

*‘When he said it, I actually started saying “that's an old man's disease”. I only got the word “that” out of my mouth and then shut up because I'm sixty. I think of myself as forty but I'm not, I'm sixty.’*

(AQ062, Male, 60 years old)

One participant resisted this narrative, feeling they were not ‘old enough’ to have shingles:

*‘And I went from thinking “oh, this is an old person's thing” because I was quite upset when I got it, to be honest. I was like “I'm not that old! I'm really not that old!”’*

(AQ061, Female, 57 years old)

#### The stress narrative.

Shingles is caused by a dormant virus, and some participants understood this and felt that physical ill health had triggered it:

*‘When I got it, I was thinking where did I get it from and things like this. But now I understand it’s dormant in your body … My immune system was down low. I wasn’t feeling one hundred per cent, I have, a few months ago, got over or given the all-clear at the moment for prostate cancer.’*

(AQ042, Male, 61 years old)

When reflecting on why they had experienced shingles, participants often linked it not only to ill physical health but also to poor mental health caused by challenging life events (or both):

*‘If I had to make a choice, I'd say stress. I'm having a very tough time with my father-in-law … So, we were having a bit of a tough time. I was also having some cashflow issues.’*

(AQ062, Male, 60 years old)

One participant even resigned from his job as a result:

*‘I thought I’d probably got this through stress. I need to make some changes, which I have, ’cause I’ve resigned from my job.’*

(AQ045, Male, 63 years old)

Healthcare professionals reinforced the perceived link between shingles and mental wellbeing:

*‘I did ask the nurse that. She said, “We’ve all got it. If we’ve had chickenpox, we all carry the virus. It can just trigger off when you’re low, or not feeling too good.” So, I think I was feeling quite low. I think maybe that was it. I think it was more to do with the season.’*

(AQ050, Male, 64 years old)

The stress narrative can lead to a sense of shame and self-blame, with participants feeling as though they could have avoided shingles by acting or feeling differently:

*‘At the time, I was really, really stressed. My mother is ninety-two, and I was looking after her … It was something that I could have avoided if I hadn’t allowed myself to get so uptight.’*

(AQ049, Female, 64 years old)

*‘I was emotionally a bit low I think, so yeah, I felt I’d not brought it on myself of course, but I got so stressed about my mother-in-law and it was on the back of other stuff, and it was like another thing on top of it.’*

(AQ048, Female, 59 years old)

One participant said he did not disclose his diagnosis to others because it suggested he was struggling with his mental and physical health:

*‘Silly thing to say, but I didn’t want to go and tell everyone I had shingles … You go online and have a look at it and think people are down in the dumps and kind of things like that, and I never considered myself anything like that … I didn’t want to own up to anything like that, and I don’t think I was.’*

(AQ041, Male, 64 years old)

### Impact on everyday life

#### Effect on sleep.

Participants reported a range of symptoms and the impact of these on everyday life. A key problem reported by participants was difficulty sleeping, because of shingles pain or irritation:

*‘I couldn’t sleep, I couldn’t get comfortable laying down, kept turning and yeah, I couldn’t get* [a] *good night’s sleep really and you couldn’t lay comfortably, moving about and that. But I don’t think it was severe. I had a couple of weeks bad sleeping really.’*

(AQ041, Male, 64 years old)

#### Limiting social and recreational activities.

Interviewees described that they limited their everyday activities, because of pain or tiredness, and also because of concerns about it being contagious:

*‘I didn't go outdoors for three weeks, I just stayed at home. I was finding it hard to get dressed, not to have anything touching it.’*

(AQ060, Female, 59 years old)

*‘The thing was that you can give other people who have never had chickenpox before, you can give them chickenpox. So I couldn’t go and see our grandson.’*

(AQ049, Female, 64 years old)

For a few participants, the visible signs of shingles also inhibited their activities or caused self-consciousness:

*‘I just limited the time that I went out … Because you could really see it because it was sort of, it was down from my cheek, down my neck.’*

(AQ059, Male, 56 years old)

#### Mental wellbeing.

A number of participants reported an impact on their mood or emotional and psychological wellbeing as a consequence of shingles. Several factors contributed to this, including the experience of symptoms such as tiredness, sense of isolation, and boredom, and a sense of not feeling their normal healthy selves:

*‘I did not go out at all for that time and that was hard for me, really hard. My mood changed a lot. I got very down, very depressed with it all.’*

(AQ061, Female, 57 years old)

#### Not yet back to normal.

Some participants reported ongoing effects and a sense that they were not yet fully recovered:

*‘Although I’m sort of recovered from shingles … I feel like I haven’t shrugged it off yet … I’m still doing all the things I was doing before but not really 100%.’*

(AQ048, Female, 59 years old)

*‘You get a little flare-up on your skin you think, “Oh my god is that the shingles and has it come back again?” … So you kind of don't trust your own body for a while.’*

(AQ061, Female, 57 years old)

## Discussion

### Summary

Shingles can be a challenging diagnosis, with patients having to cope with uncertainty about the extent to which their lives would be affected. Many people understood it was connected to the chickenpox virus, but they searched for triggers in their everyday life to understand why it had affected them, often linking it to ‘stress’ or feeling ‘low’. This was problematic as some blamed themselves or felt ashamed. Shingles affected participants’ mood and mental wellbeing, particularly if they had to make changes to their social and recreational activities. People experienced a variety of prodromal symptoms before the appearance of the rash, and some felt they were still struggling to feel returned to normal. Shingles vaccination campaigns helped participants to recognise shingles. However, those who were vaccinated felt upset and confused that they had still developed shingles.

### Strengths and limitations

To our knowledge, this is the first qualitative study in the UK about patients’ experiences and understanding of shingles. Our analysis extends the previously limited worldwide literature on lay understanding and experience of the condition, with novel insights into patients’ understanding and interaction with their social and psychological lives. Previous qualitative studies have tended to interview people long after their diagnosis and who experienced post-herpetic neuralgia, but we interviewed participants within 3 months of their shingles diagnosis, and included patients with a range of pain experiences, focusing on their understanding.

One limitation is lack of ethnic diversity, and future research could explore meanings of shingles in different communities. The perspectives of younger people are missing from our analysis (although this reflects the distribution of the disease burden). Participants had to meet the ATHENA trial eligibility criteria and therefore the perspectives of those with a delayed diagnosis, or those who were not willing/able to take part in the clinical trial, are not included. Participants’ experiences could have been affected by the study medication — benefits or harms from taking amitriptyline or placebo/nocebo effects.

### Comparison with existing literature

Understanding of shingles as a sign of ageing and of decreasing immunity found in our study has been reported previously.^7^ We suggest that the theory of biographical disruption^13^^,^^14^ can be helpful in understanding the lived experience of shingles. Participants reflected on their self-identity and came to think of themselves as ageing individuals. Further development of Bury’s theory of biographical disruption has included the idea of ‘biographical flow’^15^ to describe how individuals, when faced with an illness which alters their idea of themselves and their everyday lives, have to reconstruct a narrative about their life by incorporating the new condition. A diagnosis of shingles may cause people to reassess their everyday lives. The most extreme example in our study was the participant who had resigned from his job. The participants in our study sometimes made sense of having shingles by understanding themselves now as ageing, in a way they may not have done previously. In this way, shingles can be integrated into a person’s life (biographical flow).

Similar to previous research,^6^^,^^7^ participants in our study had limited knowledge about shingles and its presentation, progression, and complications, although most had been at least aware of it as an illness. A large international survey found that people with no previous experience of shingles were much less likely than those with previous experience to consider pain to be one of the worst symptoms, suggesting a widespread lack of understanding that shingles can be painful.^16^ One of the concepts elicited in Van Oorsocht *et al*’s study^8^ was ‘surprise’ in relation to the severity of symptoms. Our data fit this pattern, with participants feeling surprised at the extent of pain they experienced. Additionally, our analysis showed that participants looked to others’ experiences of shingles to understand how it would affect them, but they found little consistency, and prognostic uncertainty was a source of concern for participants.

A novel theme in our analysis was of participants attributing their diagnosis of shingles to ‘low’ mental wellbeing because of, for example, caring responsibilities, work stress, or the season. Participants reported that healthcare professionals reinforced the idea that shingles can be triggered when people feel ‘down’. The results of a Danish prospective cohort study support a causal relationship between high psychological stress and shingles; it reported that scoring >18 (out of 40) on a psychological stress score was associated with a 14% increase in shingles diagnosis.^17^ Regarding depression and shingles, however, existing evidence on a causal relationship is mixed. Some studies have found an association between self-reported depression and shingles,^18^^,^^19^ but a 2022 large study using UK electronic health data found no association between shingles and mental health conditions.^20^ Shingles may result in anxiety or depressive symptoms, especially in those with post-herpetic neuralgia,^3^^,^^21^ but depression is not known to be a cause of shingles.

These findings in relation to stress, mental health, and shingles are important. Patients may conflate low mood with low immunity. While low immunity is associated with shingles, any link with stress is an association rather than causally proven, and the relevance of mental health is even less clear. However, such explanations and narratives can lead to people blaming themselves and instigating life changes with far reaching consequences. One interviewee told us he was ashamed to tell others he had shingles, fearing judgement about his mental wellbeing. Research shows that men are particularly susceptible to shame around mental illness, and importantly that this can inhibit help seeking in this group.^22^^,^^23^ Some participants made changes in their lives, even retiring from work.

### Implications for research and clinical practice

Future research may usefully explore how patients’ experiences with delayed diagnoses and outside of a trial setting compare. It would also be helpful to explore the causal relationship between shingles, stress, and mental wellbeing. In the meantime, healthcare professionals and public health campaigns should ensure that patients understand that they should not blame themselves for their illness or feel ashamed. This work highlights how people experience shingles as an illness that intersects with a wide range of social and biographical aspects of their lives. It raises questions for them about their age, lifestyle, and mental wellbeing. In their interactions with patients, it is important that GPs and healthcare professionals recognise these potential sensitivities. Uncertainties regarding causation and prognosis should be acknowledged in order to moderate the impact of the condition on patients’ lives.
